# Quality of Life in Patients with Posterior Segment Eye Diseases

**DOI:** 10.18502/jovr.v21.18249

**Published:** 2026-05-24

**Authors:** Masoud Khorrami-Nejad, Foroozan Narooie-Noori, Qasim Al-khafaji, Hesam Hashemian, Shafi Balal, Thomas Dohlman, Sheraz Daya

**Affiliations:** ^1^Optometry Department, School of Rehabilitation, Tehran University of Medical Sciences, Tehran, Iran; ^2^Translational Ophthalmology Research Center, Farabi Eye Hospital, Tehran University of Medical Sciences, Tehran, Iran; ^3^Students' Scientific Research Center, Tehran University of Medical Sciences, Tehran, Iran; ^4^Moorfields Eye Hospital, London, UK; ^5^Department of Ophthalmology, Massachusetts Eye and Ear Infirmary, Harvard Medical School, Boston, MA, USA; ^6^Centre for Sight, East Grinstead, West Sussex, UK

**Keywords:** Macular Degeneration, Optic Neuropathy, Quality of Life, Posterior Segment, Retinal Diseases

## Abstract

Posterior segment eye diseases pose a significant threat to vision-related quality of life, affecting functional independence, psychological health, and social well-being. Conditions such as diabetic retinopathy and age-related macular degeneration are leading causes of blindness, often resulting in severe emotional distress and reduced mobility. Optic neuritis and optic neuropathy can lead to vision loss and increase the risk of depression, particularly in patients with multiple sclerosis. Retinal disorders like central serous chorioretinopathy and retinitis pigmentosa disrupt daily tasks such as reading and driving, thus leading to social isolation and economic burden. Hypertensive retinopathy and retinal detachment further contribute to vision impairment, with long-term implications for functional ability and mental health. Inherited retinal diseases such as Stargardt disease and Leber congenital amaurosis significantly reduce quality of life from an early age and negatively affect education, employment opportunities, and psychological well-being. Additionally, the economic impact of these diseases is substantial, with high treatment costs and productivity loss. On the other hand, vision rehabilitation, psychological support, and advancements in gene and pharmacological therapy can largely mitigate the adverse effects on quality of life associated with posterior segment diseases. Therefore, a comprehensive approach that integrates medical, psychological, and social interventions is essential for improving patient outcomes. Future research should focus on developing innovative treatments and patient-centered care strategies to improve the quality of life for individuals affected by this condition.

##  INTRODUCTION 

Posterior segment diseases are among the leading causes of progressive vision loss and blindness, significantly affecting patients' quality of life (QoL) and daily functioning. Eye diseases such as diabetic retinopathy (DR), age-related macular degeneration (AMD), optic neuropathy, and inherited retinal diseases often result in irreversible visual impairment, leading to reduced independence, emotional distress, and occupational limitations.^[1,2,3]^ Many of these diseases have a chronic and progressive nature that makes their long-term management challenging and further exacerbates their impact on overall well-being.

The functional effects of posterior segment diseases extend beyond visual acuity loss. Patients frequently report difficulty with mobility, reading, facial recognition, and depth perception, which directly impact their ability to maintain employment and engagement in social activities. Additionally, psychological distress, including depression and anxiety, is prevalent among individuals with vision-threatening retinal disorders.^[1,2,3]^ The financial burden posed by these diseases is also considerable, and high costs are associated with frequent medical visits, treatments such as anti-VEGF injections, and assistive technologies required for daily living.^[1,2,3]^

Despite advancements in ophthalmic care, many posterior segment diseases remain difficult to treat. This challenge necessitates a multidisciplinary approach that integrates medical, psychological, and rehabilitative strategies to improve QoL. The purpose of this review is to examine the impact of posterior segment diseases on QoL and explore their functional, psychological, and economic consequences while discussing current and emerging strategies for improving patient outcomes.

##  METHODS

The literature for this review paper was collected through a comprehensive search of peer-reviewed articles, clinical studies, and relevant publications focusing on posterior segment eye diseases and their impact on QoL. Databases such as PubMed, Scopus, Google Scholar, and Web of Science were utilized to identify studies published in English between 2002 and 2024. Keywords such as “quality of life”, “posterior segment diseases”, “retinal disorders”, “optic neuritis”, “optic neuropathy”, “age-related macular degeneration”, “birdshot chorioretinopathy”, “central serous chorioretinopathy”, “retinoschisis”, “diabetic macular edema”, “diabetic retinopathy”, “hypertensive retinopathy”, “retinopathy of prematurity”, “retinal detachment”, “retinitis pigmentosa”, “Stargardt disease”, “inherited retinal diseases”, “Leber congenital amaurosis”, “Best disease”, “choroideremia”, “juvenile x-linked retinoschisis”, “cone-rod dystrophies” and “night blindness” were combined with Boolean operators (AND, OR) to refine the search results. Articles were screened based on their relevance to the physical, psychological, social, and economic implications of posterior segment eye diseases. References from selected articles were also reviewed to ensure comprehensive coverage. Only studies providing original data, validated QoL measurements, and detailed methodologies were included to maintain the reliability of the present review.

### Optic Neuritis 

Optic neuritis (ON) significantly impacts patients' QoL, particularly in terms of visual function and psychological well-being.^[4,5,6]^ While many patients recover their visual acuity, they often continue to experience lasting impairments in contrast sensitivity, visual fields, and daily visual tasks. Approximately 72% of affected eyes achieve 20/20 visual acuity after 15 years, but subtle deficits in visual function persist, especially in patients with multiple sclerosis (MS), leading to reduced QoL scores on assessments like the National Eye Institute Visual Function Questionnaire-25 (NEI VFQ-25).^[4]^

In atypical ON, which is often more severe than the typical form, vision-related QoL is particularly affected by bilateral involvement and severe visual impairment. Patients with binocular disease report lower composite NEI VFQ-39 scores, especially in subscales related to role difficulties, dependency, and mental health. Furthermore, the best-corrected visual acuity (BCVA) in the better-seeing eye strongly correlates with QoL, which underscores the importance of preserving vision in at least one eye. Patients with BCVA poorer than 20/200 in their better-seeing eye experience the greatest decline in QoL, highlighting the need for timely and effective treatment to prevent severe vision loss.^[5]^

Additionally, the psychological burden of ON cannot be overlooked. Even patients with near-normal visual acuity may perceive significant declines in QoL due to visual disturbances, anxiety about disease progression, and the potential association with MS. This is evident in the lower QoL scores reported for patients with neurologic disability from MS compared to those without MS.^[4]^ Comprehensive care addressing both visual and emotional challenges is essential to improving QoL in patients with ON.

### Ischemic Optic Neuropathy 

Optic neuropathy significantly impacts patients' QoL, particularly in terms of visual function and daily activities. Patients experience substantial visual decline as a result of non-arteritic anterior ischemic optic neuropathy (NAION). The condition leads to reduced visual acuity, visual field defects, and changes in retinal nerve fiber layer (RNFL) thickness and vessel density (VD). These impairments correlate strongly with reduced vision-related QoL (VR-QoL). Patients with NAION report significantly lower scores on the NEI-VFQ-25 across most subscales, particularly in areas like driving, role difficulties, and social functioning. These findings highlight how deeply NAION affects both functional vision and psychological well-being. Retinal parameters such as RNFL and VD also show significant correlations with QoL metrics, underscoring the importance of retinal health in maintaining QoL.^[6]^

Rehabilitation strategies, such as vision restoration therapy (VRT), have shown promise in improving visual function and QoL in patients with optic neuropathy. A pilot study on anterior ischemic optic neuropathy (AION) demonstrated that targeted stimulation of residual vision areas (ARV) could lead to improvements in binocular reading speed and foveal sensitivity. Patients also reported functional improvements in daily activities, suggesting that visual rehabilitation can positively influence QoL. However, further studies are needed to confirm these findings and refine treatment strategies.^[2]^ By integrating both the visual and psychological aspects of ischemic optic neuropathy, comprehensive management approaches can help improve QoL for affected individuals.

### Age-related Macular Degeneration (AMD) 

AMD is one of the leading causes of irreversible vision loss in older adults and significantly impacts their QoL.^[3,7,8,9,10]^ The condition affects central vision, which is crucial for performing daily activities such as reading, driving, and recognizing faces, thereby posing physical, emotional, and social challenges for the patients. Emphasizing the need for comprehensive management approaches, studies have consistently highlighted the profound effect of AMD on VR-QoL.

Patients with AMD often experience difficulties in performing daily activities, emotional distress, and reduced independence. For instance, AMD has been associated with diminished physical functioning and general health, as measured by tools like the SF-36, with significant reductions in physical and social functioning subscale scores.^[7]^ This impairment often necessitates assistance with daily activities, which further reduces autonomy. Moreover, AMD's impact on QoL is not limited to physical challenges. Thus, depressive symptoms are so prevalent that nearly half of patients with AMD exhibit clinically significant depression, compared to 17.5% in age-matched controls. Regarding the multidimensional impact of the condition, path analyses suggest that AMD contributes to depressive symptoms directly and indirectly by reducing general and social functioning.^[7]^

QoL is further influenced by the severity of the disease. For example, patients with advanced AMD, particularly those with bilateral involvement, often report a greater reduction in VR-QoL than those with milder forms of the disease.^[8]^ Tools like the VF-14 and NEI-VFQ-25 have been instrumental in assessing the functional limitations of patients with AMD, revealing that AMD severity correlates significantly with reduced scores in areas like reading, driving, and mental health.^[8,9]^

Another critical issue concerns the disparity between patients' and clinicians' perceptions of the impact of AMD on QoL. Ophthalmologists often underestimate the severity of the condition's effect on patients' lives. For instance, a study comparing utilities derived from patients with those from ophthalmologists revealed that physicians consistently undervalued the burden of AMD. Patients with severe vision loss expressed the willingness to trade a significantly greater proportion of their remaining years for perfect vision, reflecting the profound impact on their perceived QoL.^[3]^

Treatment interventions, particularly anti-VEGF therapies, have shown promise in stabilizing or improving vision in patients with neovascular AMD. However, the burden of frequent injections and follow-ups can further affect QoL. The EQUADE (QoL in patients suffering from active exudative age-related macular degeneration) study has demonstrated that while anti-VEGF treatments improve visual outcomes, the associated healthcare and social care burdens significantly influence QoL, particularly in domains like mental health and dependency.^[10]^ Patients often require assistance with transportation, household tasks, and other daily activities, which can exacerbate feelings of dependency and negatively impact their overall well-being.

Despite these challenges, some patients with AMD report good life satisfaction, often relying on adaptive strategies and support systems to cope with the limitations posed by the disease. However, these coping mechanisms are not universal, and many patients experience severe reductions in QoL, particularly in advanced stages of AMD.^[7,9]^

In conclusion, AMD significantly affects QoL across physical, psychological, and social domains. The VR-QoL is reduced by the severity of the disease, with advanced stages causing profound impairments. Tools like the NEI-VFQ-25, VF-14, and MacDQoL have been invaluable in assessing the condition's impact, guiding clinicians in understanding patients' experiences. Addressing the physical and emotional needs of patients with AMD through early intervention, patient education, and psychological support is crucial for improving QoL. Future research should focus on refining treatment strategies and developing interventions that address the multifaceted challenges faced by this population.

### Birdshot Chorioretinopathy 

Birdshot chorioretinopathy (BSCR) significantly impairs patients' QoL due to its effects on vision, emotional well-being, and physical independence. Despite often preserving good central visual acuity, patients with BSCR experience severe functional limitations, including reduced contrast sensitivity, nyctalopia, and visual field loss.^[11]^

NEI VFQ-25 has provided critical insights into the disease's impact on vision-specific QoL. Levinson et al found a median composite score of 76.8, with significant reductions in subscales such as mental health, dependency, and driving. Nyctalopia was particularly associated with lower scores, emphasizing its role in diminishing QoL.^[12]^ Similarly, Kaisari et al in 2024 reported a mean composite score of 58 ± 30 in elderly patients with BSCR, with driving and mental health among the most affected subscales.^[13]^ These findings highlight the extensive functional and emotional burden of the disease.

Contrast sensitivity deficits are a hallmark of BSCR and further worsen QoL. Kappel et al reported that, even in patients with normal BCVA, 92% of eyes had abnormal contrast sensitivity at 12 cycles per degree.^[11]^ Impaired contrast sensitivity strongly correlated with lower NEI VFQ-25 scores, particularly in near vision and driving subscales—underscoring its functional significance.

The psychological impact of BSCR is profound, as the threat of progressive vision loss leads to anxiety, depression, and reduced independence. Regarding the emotional burden of BSCR, Levinson et al noted that mental health consistently scored the lowest among NEI VFQ-25 subscales.^[12]^ Dependency and social functioning are also heavily impacted, particularly in patients with severe visual impairments.^[13]^

Functional impairments, such as difficulties with driving and mobility, are among the most significant QoL challenges in patients with BSCR. Peripheral vision deficits increase reliance on others, thereby limiting independence and social participation.^[12,14]^

In conclusion, BSCR considerably affects QoL by impairing vision, mental health, and independence. Tools like the NEI VFQ-25 and contrast sensitivity assessments provide valuable insights into its broader impact. Managing BSCR requires a holistic approach that addresses not only visual outcomes but also the emotional and social dimensions of the disease.

### Central Serous Chorioretinopathy 

Central serous chorioretinopathy (CSCR) significantly impacts patients' QoL primarily by impairing vision, which is usually associated with psychological stress. Patients with either acute or chronic forms of CSCR often report reduced VR-QoL due to symptoms such as micropsia, metamorphopsia, central scotoma, and decreased visual acuity, which interfere with daily activities and social functioning.^[15]^ Using the NEI VFQ-25, studies have consistently shown lower scores in QoL metrics for patients with CSCR compared to healthy controls. Patients with chronic CSCR often report worse outcomes than those with acute forms of the disease.^[16]^

The psychological burden of CSCR is also notable. Elevated stress levels, depressive symptoms, and anxiety are common in patients with CSCR, particularly during the acute phase of the disease.^[17]^ As a recognized risk factor for CSCR, stress may exacerbate disease symptoms and contribute to a cycle of psychological distress and visual impairment.^[18]^ However, it is unclear whether stress is a cause or a consequence of the disease, given that stress levels tend to normalize during follow-up periods.^[17]^

Chronic CSCR, in particular, is associated with long-term decline in VR-QoL. Breukink et al demonstrated that patients with chronic CSCR experienced significant visual acuity loss over time, accompanied by lower scores on multiple domains of the NEI VFQ, such as social functioning, mental health, and role difficulties.^[15]^ Additionally, patients with persistent subretinal fluid often report prolonged visual disturbances, further reducing QoL.^[19]^

Interestingly, some studies suggest that the impact of CSCR on QoL may be less severe compared to other maculopathies such as DR or ARMD. The younger age and transient nature of acute CSCR symptoms may partly explain this difference.^[15,16]^ Nevertheless, the progressive nature of chronic CSCR and its psychological implications emphasize the need for timely intervention and psychosocial support to improve patients' overall well-being. Psychological counseling and stress management strategies may be beneficial in mitigating the broader impact of CSCR on QoL.^[18]^

### Juvenile Retinoschisis 

X-linked retinoschisis (XLRS) significantly affects patients' QoL due to its effects on vision and the resulting challenges in daily functioning. Patients with XLRS often experience reduced visual acuity, visual field defects, and complications such as retinal detachment, which collectively contribute to difficulties in performing routine activities, including reading and driving.^[20]^ Regarding the broad impact of XLRS on QoL, studies using the NEI VFQ-25 scale demonstrate that affected individuals report lower scores in vision-specific domains like near and distance activities, social functioning, and mental health.^[20]^

For parents of children with XLRS, the condition also presents emotional and psychosocial challenges. Parents report stress and anxiety stemming from their children's uncertain visual prognosis, as well as challenges in balancing the patient's need for protection with promoting independence in activities like sports and driving.^[21]^ These findings emphasize the need for comprehensive care, including psychosocial support and individualized strategies, to help patients and families cope with the condition's long-term implications.

### Diabetic Macular Edema 

Diabetic macular edema (DME) significantly impacts patients' QoL, particularly in terms of vision-related functioning and overall well-being. Studies have shown that DME leads to a substantial decline in VR-QoL.^[22]^ Patients with DME often report difficulties performing daily activities, such as reading, driving, and recognizing faces, which are essential for maintaining independence and engaging in social interactions.^[22]^ DME is also associated with emotional and psychological burden, such that many patients experience anxiety, depression, and a sense of dependency on others due to their visual impairment.^[22]^

The economic burden of DME exacerbates the other challenges faced by patients. Direct medical costs—including frequent treatments and hospital visits—alongside indirect costs such as lost productivity, contribute to the overall financial strain.^[23]^ Anti-VEGF therapies have been shown to improve both visual acuity and VR-QoL, offering patients a better QoL compared to traditional treatments like laser therapy.^[24]^ However, the high cost of these treatments and the need for repeated injections can be a significant burden for patients and healthcare systems alike.^[23]^

Comparative studies have suggested that VR-QoL in patients with DME is often similar to that of patients with AMD.^[22]^ However, patients with DME tend to report lower QoL scores compared to those with other ocular conditions like glaucoma or cataracts, underscoring the severe impact of DME on daily functioning.^[22]^ Patient-reported outcomes, such as those captured by the NEI VFQ-25, are increasingly recognized as essential in guiding treatment decisions and evaluating the effectiveness of interventions.^[24]^

In conclusion, DME affects not only patients' visual acuity but also their overall QoL, highlighting the need for effective and accessible treatments to mitigate these adverse effects.

### Diabetic Retinopathy (DR) 

DR is a significant microvascular complication of diabetes and is widely recognized as a major factor affecting QoL in patients with diabetes due to its direct impact on vision-related and overall health-related QoL (HRQoL).^[3,25,26,27,28,29]^ Studies have shown that DR, particularly in its advanced stages, is associated with a marked reduction in HRQoL across various domains, including physical, emotional, and social well-being.^[28,30,31,32]^

Several investigations have highlighted the profound impact of DR on HRQoL. Pan et al demonstrated that Chinese patients with bilateral DR reported lower EQ-5D health utility scores compared to those without DR, particularly in domains related to routine activities.^[28]^ This finding underscores the significant functional limitations imposed by DR, even in community-dwelling individuals. Similarly, Alcubierre et al found that DR significantly reduced QoL in patients with type 2 diabetes, with the most significant impact observed in physical ability, personal relationships, sex life, motivation, future, dependence, and leisure.^[32]^

The severity of DR also plays a critical role in determining its impact on QoL. Venkataraman et al reported that severe retinopathy was associated with significantly lower scores in HRQoL domains such as physical functioning, role limitations due to physical problems, and social functioning. This observation confirms the progressive nature of DR's impact, such that more advanced stages of the disease lead to worse HRQoL outcomes.^[30]^ Moreover, Tekle et al found that Ethiopian patients with diabetes and DR experienced substantial problems regarding pain/discomfort and mobility, further highlighting the physical burden of the condition.^[31]^

Interestingly, the impact of DR on QoL is not limited to vision-specific domains. Pan et al observed that DR was associated with anxiety and depression, contributing to a broader psychosocial burden.^[28]^ Similarly, Khashayar et al identified a strong association between DR and emotional distress, particularly in women and patients with other diabetes-related complications. The authors suggested that the psychological impact of DR extends beyond its physical manifestations.^[33]^

On the other hand, the type of DR appears to influence the degree of QoL impairment. Pan et al noted that while both unilateral and bilateral DR negatively affected QoL, the impact was more pronounced in bilateral cases, likely due to the greater functional limitations associated with vision loss in both eyes. However, the difference in QoL between unilateral and bilateral DR was not statistically significant in all domains, suggesting that even early stage DR can substantially affect patients' lives.^[28]^

The management of DR also contributes to its impact on QoL. Alcubierre et al highlighted that patients who underwent photocoagulation therapy reported lower QoL, particularly in domains related to treatment satisfaction and physical health.^[32]^ This finding suggests that while treatments may help slow disease progression, they may also introduce additional burdens, such as discomfort or limitations in daily activities.

Despite these challenges, early detection and intervention can mitigate some of the adverse effects of this condition on QoL. Huang et al emphasized the importance of comprehensive diabetes care, including regular screening and timely treatment for DR, as a means to preserve QoL.^[34]^ Similarly, Lui et al found that the residual impacts of DR on HRQoL could persist even after treatment. The researchers, therefore, underscored the need for continuous management and support for affected individuals.^[35]^

Moreover, the coexistence of DR with other complications of diabetes amplifies its impact on QoL. Venkataraman et al observed that patients with both DR and other microvascular complications, such as nephropathy and neuropathy, reported significantly lower QoL scores compared to those with DR alone.^[30]^ This suggests that the cumulative burden of multiple complications necessitates a holistic approach to diabetes management.

In conclusion, DR significantly reduces QoL in patients with diabetes, with its impact extending beyond vision-specific domains to encompass broader physical, emotional, and social dimensions. The severity and bilateral nature of DR and the presence of additional complications further exacerbate its effects. Mitigating the impact of DR on QoL and improving treatment outcomes for patients with DM requires effective management strategies such as early detection, timely intervention, and comprehensive care. Table 1 shows the HRQoL outcomes in patients with the most common posterior segment diseases.

### Hypertensive Retinopathy 

Hypertensive retinopathy (HR) significantly impacts QoL through its association with visual impairment and systemic complications.^[36,37,38]^ Studies have demonstrated that HR is not merely a localized condition but a marker of widespread vascular damage that influences both physical and emotional well-being. Porta et al highlighted that advanced HR (grades III and IV) is linked to reduced survival and increased morbidity, which indirectly affects QoL by contributing to physical limitations and psychological burden of this chronic disease.^[36]^ Retinal changes in HR, such as arteriolar narrowing and hemorrhages, signify microvascular damage that may reflect similar processes in other critical organs and further impair QoL.

Shrestha et al found that patients with HR often experience secondary complications like visual impairment, which can lead to decreased competence in daily activities and heightened family burden. Underscoring the importance of lifestyle modifications to preserve QoL, the authors reported that smokers and individuals with long-standing hypertension were more likely to develop HR. Moreover, they emphasized the need for patient education about the ocular and systemic effects of hypertension to mitigate its impact on daily life.^[39]^

Del Brutto et al identified that HR severity correlates with increased all-cause mortality in older adults, particularly in rural populations with limited health care access.^[38]^ This association confirms the broader implications of HR on general health and life expectancy, which are critical components of QoL. Early detection and systemic management of HR could reduce its detrimental effects on physical and emotional health, ultimately improving QoL for affected individuals.

### Retinopathy of Prematurity

Retinopathy of prematurity (ROP) significantly affects patients' QoL, with its impact extending to later childhood and adulthood.^[40,41,42]^ Studies have demonstrated that increasing severity of ROP is associated with greater limitations in functional domains and reduced HRQoL. For instance, children with severe ROP requiring surgical intervention at birth exhibit significantly lower QoL scores at age 10 years, particularly in areas such as physical, social, and emotional functioning.^[42]^ Similarly, the Cryotherapy for Retinopathy of Prematurity (CRYO-ROP) study revealed that children with threshold ROP had reduced HRQoL compared to those without ROP. It was suggested that severe visual impairment contributes to poorer scores across multiple attributes, including vision, dexterity, and cognition.^[40]^

The presence of visual and neurodevelopmental sequelae in children with ROP further impairs QoL. Kesarwani et al reported that children treated for ROP had significantly lower scores in domains such as family impact and treatment difficulty. Also, ocular conditions like amblyopia and strabismus in children with ROP were associated with poorer parent-reported QoL scores.^[41]^ Additionally, the Gutenberg Prematurity Eye Study highlighted that adults with a history of ROP, particularly those requiring treatment, experienced reduced VR-QoL. The authors reported lower scores on measures of visual functioning and socioemotional well-being.^[43]^

Overall, the evidence supports the profound and long-lasting impact of this ocular condition on QoL, particularly for individuals with severe disease or associated complications. It is imperative to adopt tailored interventions to prevent and treat ROP and take supportive measures to address its sequelae so that both affected individuals and their families experience better outcomes and QoL.

**Table 1:** Health-related quality of life outcomes in patients with the most common posterior segment diseases

**Disease**	**Reference**	**Year**	**QoL instrument**	**Sample size (number of patients)**	**Main findings**
Glaucoma	Wolfram et al^[80]^	2013	• HUI • NEI-VFQ-25	154	• The NEI-VFQ-25 scores highlight various aspects of quality of life (QoL), and patients with glaucoma consider reduced peripheral vision and difficulties in driving more critical than social factors.
	Riva et al^[81]^	2019	• NEI-VFQ-25 • GSS	178	• Patients in the early to moderate stages of glaucoma had higher scores on most GSS and NEI-VFQ-25 items. • Lower BCVA was linked to lower scores in 4 of the 12 NEI-VFQ-25 items.
	Majerníková et al^[82]^	2021	• WHOQOL-BREF • NEI-VFQ-25	119	• The WHOQOL-BREF assessment revealed significant differences in the psychological and environmental domains among those with better vision in one eye. • The NEI-VFQ-25 showed significant differences across all domains except for the driving subscale.
	Zhang et al^[83]^	2022	• BIPQ • GQL-15	97	• The BIPQ total score had a positive correlation with both the GQL-15 total score and its four dimensions.
	Knight et al^[84]^	2022	• Semi-structured questionnaire	47	• Coping strategies and emotional well-being were the most notable themes. • Maladaptive coping strategies, including treatment nonadherence, were more frequently observed in individuals under 40 years old, those without vision impairment, or those who attended fewer follow-ups.
	Sen et al^[85]^	2024	• WHOQOL-BREF	199	• QoL values were significantly influenced by income. • Variations in intraocular pressure affected psychological aspects of QoL. • The severity of the disease did not show a significant association with QoL.
Diabetic retinopathy (DR)	Huang et al^[34]^	2007	• Time-tradeoff questions	701	• End-stage complications, such as blindness, had lower utility scores compared to intermediate complications like retinopathy. • Among all health states, end-stage complications received the lowest ratings. • Intensive treatments had lower mean utility scores than conventional treatments, such as intensive glucose control versus conventional glucose control.
	Matza et al^[27]^	2008	• NEI-VFQ-25 • SF-36	535	• Patients who experienced a loss of > 10 letters in vision had significantly greater declines in all VFQ-25 subscales except for ocular pain, compared to those whose visual acuity remained unchanged. • Changes in SF-36 scores did not align as closely with changes in vision. • A continuous decline in visual acuity was significantly linked to declines in all VFQ-25 scales except ocular pain.
	Peasgood et al^[86]^	2016	• EQ-5D • SF-6D • EQ-VAS	2341	• Depression and diabetic foot disease led to a considerable reduction in health-related QoL (HRQoL) for patients with T1DM. • HbA1c (%) appeared to have an independent negative effect on HRQoL, though concerns remain about potential endogeneity.
	Vijayan et al^[29]^	2017	• NEI-VFQ-25	141	• Comparing pre- and post-treatment QoL indicated an overall improvement in QoL following treatment. • Overall QoL improved for all participants after treatment, with the most significant improvements observed in dependency, SF, and MH.
	Pan et al^[28]^	2018	• EQ-5D-5L	913	• Chinese patients with T2DM and bilateral DR reported lower HRQoL scores than those without DR, particularly in areas related to usual daily activities.
	Parik et al^[87]^	2019	• EQ-5D-5L	360	• The most frequently reported health issues were in the pain/discomfort category, followed by mobility problems. • Older age, poor glycemic control, longer diabetes duration, insulin use, obesity, and diabetes-related complications were significant negative predictors of HRQoL.
Age-related macular degeneration	Brody et al^[88]^	2002	• Profile of Mood States • NEI-VFQ • Duke Social Support Index • Life Optimism Test–Revised • AMD Self-Efficacy Questionnaire	231	• Patients in the self-management group showed notable improvements in mood and overall function compared to the control group. • These improvements were significantly greater in individuals with depression than those without depression. • Reduced emotional distress was linked to higher self-efficacy, while functional improvements correlated with increased self-efficacy and perceived social support.
	Berdeaux et al^[89]^	2005	• NEI-VFQ–39	114	• VA was not associated with general health or ocular pain scores. • General vision, near activities, distance vision, driving, mental health, role difficulties, dependency, peripheral vision, and overall NEI-VFQ scores were influenced by both the best and worst eye VA.
	Mathew et al^[7]^	2011	• GAD scale • SF-36	145	• Individuals with AMD performed worse than controls on the GAD depression scale and the physical functioning subscale of SF-36.
	Šiaudvytytė et al^[90]^	2012	• Visual Functioning Questionnaire • HADS scale	140	• Patients with AMD, whether affecting one or both eyes, had significant differences in near vision, distance vision, color vision, and social functioning. • In the AMD group, general vision was significantly linked to mental health, dependency, and role difficulties.
	Matamoros et al^[10]^	2015	• NEI-VFQ-25	416	• For patients with exudative AMD treated with ranibizumab, the key predictors of QoL included VA outcomes, home healthcare, and social support services.
	Vu et al^[91]^	2007	• NEI-VFQ25 • SF-36 • EuroQoL EQ-5D-5L	547	• The QoL of patients with nAMD was influenced by visual acuity, medical history, mental health, and functional status.
QoL, quality of life; HUI, Health Utility Index; NEI-VFQ, National Eye Institute Visual Functioning Questionnaire; OHT, ocular hypertension; POAG, primary open angle glaucoma; GSS, Glaucoma Symptom Scale; BCVA, best-corrected visual acuity; WHOQOL-BREF, World Health Organization Quality of Life; BIPQSF, Brief Illness Perception Questionnaire; Social Functioning; MH, mental health; GAD, Goldberg Anxiety and Depression; HADS, Hospital Anxiety and Depression Scale; GQL-15, Glaucoma Quality of Life-15; SF-36, 36-Item Short Form Survey; HRQoL, health-related quality of life; EQ-5D, EuroQol- 5 Dimension; SF-6D, Short-Form Six-Dimension; EQ-VAS, EuroQol-Visual Analogue Scales; EQ-5D-5L, EuroQol-5 Dimensions-5 Levels; DM, diabetes mellitus; nAMD, neovascular age-related macular degeneration.					

### Retinal Detachment 

Retinal detachment (RD) significantly impacts VR-QoL, with patients often experiencing persistent visual and psychological challenges despite surgical success. The degree of macular involvement and postoperative recovery heavily influence VR-QoL outcomes.^[44,45,46]^ Using the modified NEI-VFQ-25, Potic et al found that patients with macula-off RD exhibit a consistent improvement in VR-QoL post-surgery, particularly in socioemotional domains. In contrast, patients with macula-on RD experience a transient decline in VR-QoL shortly after surgery, which typically returns to baseline by three months.^[44]^ This highlights the distinct recovery trajectories between macula-on and macula-off RD, with the socioemotional subscale demonstrating the most pronounced variations.

Zou et al emphasized the long-term psychological toll of macula-off RD. They reported significant impairments in VR-QoL, particularly in psychological adjustment and detailed visual tasks such as reading and recognizing faces.^[47]^ Similarly, Okamoto et al observed that even after successful RD surgeries, patients reported lower VR-QoL compared to age-matched controls. Notably, subscales related to mental health and dependency were most affected, underscoring the broader psychosocial impact of RD.^[45]^ Additionally, Mozaffarieh et al documented high levels of anxiety and depression in patients with RD both pre- and post-surgery, with psychological distress correlating strongly with visual acuity outcomes.^[46]^

Visual function deficits play a central role in reduced QoL. Garcia et al demonstrated that posterior vitreous detachment, a precursor to RD, leads to significant reductions in contrast sensitivity function, even in the absence of retinal pathology.^[48]^ This degradation in visual function negatively affects patients' daily activities and reduces their satisfaction with their visual outcomes. Fukuda et al highlighted the role of metamorphopsia in diminished VR-QoL and showed that persistent visual distortions after RD surgery are strongly correlated with lower socioemotional well-being.^[49]^

Patient expectations also influence satisfaction with surgical outcomes. Singh et al reported that dissatisfaction often arises from unrealistic preoperative expectations, even when objective visual improvements are achieved. According to these authors, dissatisfied patients typically had higher initial expectations of visual recovery and lower subjective ratings of postoperative improvement, despite similar gains in visual acuity compared to satisfied patients.^[50]^ These findings emphasize the importance of managing patient expectations through preoperative counseling.

Highlighting the broader implications of RD-related symptoms, Sebag suggested that floaters and other visual disturbances significantly impair utility values—a measure of QoL. He equated the impact of these symptoms to those of chronic systemic conditions such as diabetes or mild angina.^[51]^ Wagle et al quantified this further and showed that patients with RD-related symptoms were willing to trade life expectancy to alleviate their symptoms. This stark willingness highlights the significant impact of visual disturbances on daily life and overall well-being.^[52]^

Yannuzzi et al emphasized the cost-utility of timely RD evaluations and prophylactic treatments, noting that early detection and management of retinal tears can prevent severe visual loss and improve VR-QoL. Their findings support that screening and treating RD-related precursors are cost-effective and offer substantial benefits in preserving vision and QoL.^[53]^

Collectively, these studies highlight the multifaceted impact of RD on VR-QoL that encompasses both visual function and psychological well-being. While patients with macula-off RD demonstrate steady postoperative improvements, residual challenges such as visual distortions, anxiety, and unmet expectations remain significant barriers to fully restoring QoL. Comprehensive management, including timely intervention, meticulous preoperative counseling, and addressing postoperative concerns, is crucial to optimizing VR-QoL outcomes for patients with RD.

#### Retinitis Pigmentosa 

Retinitis pigmentosa (RP) significantly affects various aspects of an individual's QOL, including vision-related functionality, mental health, and socioeconomic factors.^[54,55,56,57,58,59,60,61,62]^ Studies consistently highlight the correlation between declining visual function and reduced QoL scores. For instance, Chaumet-Riffaud et al found that visual acuity and residual visual fields are strong predictors of self-reported QoL. The authors added that severe visual field constriction (below 10 degrees) leads to substantial limitations in mobility and social interactions.^[59]^ Similarly, Sugawara et al demonstrated a significant negative correlation between peripheral visual field loss and QoL, emphasizing the substantial impact of visual field constriction on daily activities and independence.^[60]^

Mental health challenges are prevalent among individuals with RP and often compound the effects of vision loss. Humphries et al reported that 47.4% of patients with RP in their study experienced depressive symptoms, with those having a BCVA worse than 20/200 being significantly more likely to report depression.^[54]^ Likewise, Chaumet-Riffaud et al observed higher rates of anxiety (36.5%) and depression (15.5%) among patients with RP compared to the general population.^[59]^ Meanwhile, Chaumet-Riffaud et al clarified that depression was more common in individuals with worse visual function, although its prevalence did not consistently increase with disability levels.^[59]^

Socioeconomic factors further influence QoL in patients with RP. Watanabe et al highlighted the economic burden of RP on Japanese patients, noting substantial lifetime costs associated with visual impairment, including out-of-pocket expenses for vision aids and reduced work productivity.^[63]^ Employment is particularly affected, with Levinson et al noting that only 46.5% of patients with RP in their study were employed. The authors concluded that employment status was directly associated with better self-reported QoL and lower levels of disability.^[58]^ Chaumet-Riffaud et al also emphasized the role of workplace accommodations and support in sustaining employment, particularly for individuals with severe visual disabilities.^[59]^

Physical activity has emerged as a potential modifiable factor influencing QoL in RP. Using the NEI VFQ-25, Levinson et al found that patients engaging in higher levels of physical activity reported better VR-QoL scores, particularly in peripheral and color vision. These findings suggest that lifestyle modifications, such as exercise, may have a positive impact on visual function and overall well-being.^[58]^

Education and social support are additional determinants of QoL. Prem Senthil et al noted that individuals with RP face challenges in daily activities, social participation, and emotional well-being due to progressive vision loss. However, those with strong social networks and access to adaptive resources demonstrated better coping mechanisms and resilience.^[64]^

Collectively, these studies underscore the multifaceted impact of RP on QoL. They emphasize the need for comprehensive management strategies that address visual function, mental health, socioeconomic challenges, and lifestyle interventions to improve outcomes and support for individuals living with RP.

### Stargardt Disease 

Stargardt disease (SD) significantly impacts QoL due to its progressive central vision loss, which interferes with daily activities such as reading, recognizing faces, and navigating unfamiliar environments.^[65,66,67]^ Moschos et al highlighted that patients with SD experience moderate depressive symptoms associated with declining visual function, as indicated by elevated PHQ-9 and Zung scores.^[68]^ Their study illustrated the complex interplay between vision loss and mental health and emphasized that individuals with lower BCVA reported more depressive symptoms.^[68]^ Gomes et al found that patients with SD had significantly lower QoL scores (mean NEI VFQ-25 score of 59.7) compared to healthy controls with a mean score of 88.7. This reduction in QoL was strongly correlated with depressive symptoms, as measured by both the Beck Depression Inventory and the Hamilton Depression Rating Scale.^[66]^ These findings suggest that depression is a critical factor influencing QoL independent of sociodemographic factors.

Murro et al further demonstrated that reading performance, particularly maximum reading speed and reading acuity, strongly correlates with QoL in patients with SD. They proposed that central vision loss, a hallmark of SD, is a primary factor affecting both reading ability and QoL. Moreover, the authors emphasized the importance of low vision rehabilitation for improving reading performance and overall QoL.^[67]^

Anastasakis et al noted that patients with SD often achieve high educational levels despite significant vision loss, suggesting that these individuals have developed effective coping strategies and adjustments. However, they also highlighted the emotional challenges associated with the disease, which can influence perceptions of QoL.^[69]^ Collectively, these studies underscore the need for comprehensive support, including mental healthcare and vision rehabilitation, to enhance QoL in this group of patients.

### Other Inherited Retinal Diseases 

Inherited retinal diseases (IRDs) beyond retinitis pigmentosa and Stargardt disease pose significant challenges to patients' QoL and negatively affect their physical, mental, and social well-being. Conditions such as cone-rod dystrophies,^[70,71,72]^ choroideremia,^[73]^ Leber congenital amaurosis (LCA),^[74,75]^ and Best disease^[70]^ demonstrate unique characteristics that influence patients' daily functioning and emotional health.

#### Leber congenital amaurosis (LCA) 

LCA is a severe retinal dystrophy that manifests in early childhood and presents unique QoL challenges due to its profound visual impairment from infancy.^[74,75]^ Highlighting the psychological toll of LCA, Yioti et al noted increased levels of phobic anxiety and paranoid ideation among affected individuals. These psychiatric manifestations were linked to the disease's hereditary nature and the associated fear of blindness in both patients and their families. Additionally, the authors found that illness perceptions, such as concerns about disease consequences and timeline, were strongly associated with psychological distress and lower satisfaction with general health.^[72]^

#### Best disease 

Best disease and other forms of macular dystrophies, which predominantly affect central vision, also significantly impair QoL. Karuntu et al emphasized that patients with CRB1-associated retinal dystrophies, including macular dystrophies, experience a notable decline in VR-QoL over time. Using the NEI VFQ-39, they found that the most affected domains were near activities and general vision, which is consistent with the progressive nature of macular damage. Interestingly, the decline in QoL was most pronounced during the early stages of follow-up, suggesting that patients may initially struggle to adapt to vision loss before developing coping mechanisms.^[70]^

#### Choroideremia

A rare X-linked retinal dystrophy, choroideremia primarily affects males and results in progressive peripheral vision loss. Prem Senthil et al developed psychometrically valid item banks to assess QoL across multiple domains for hereditary retinal diseases like choroideremia. They found that activity limitation, mobility, and emotional well-being were the most affected domains for patients with peripheral vision loss. Using computerized adaptive testing (CAT), the authors demonstrated that targeted assessment tools could efficiently capture the nuanced QoL effects of choroideremia, emphasizing the need for disease-specific measures.^[73]^

#### Cone-rod dystrophies 

Cone-rod dystrophies are marked by progressive central and peripheral vision loss and are associated with substantial impairment in QoL. McGuinness et al reported that individuals with cone-rod dystrophies experience lower health utility scores compared to those with central retinal diseases, which highlights the greater impact of peripheral vision loss on mobility and daily activities. The EQ-5D-5L scores, which assess general health, revealed significant challenges in mobility and anxiety/depression dimensions for these patients. Moreover, the associated VR-QoL, as measured by the NEI-VFQ-25, showed moderate correlations with general health utilities, suggesting that vision-specific impairments heavily influence overall health perceptions.^[76]^

#### Night blindness 

Night blindness significantly impacts QoL through limiting mobility, independence, and social engagement, especially under low-light conditions. Patients with congenital stationary night blindness (CSNB), for instance, encounter specific challenges depending on the severity of their condition. Bijveld et al reported that patients with CSNB1 experience more pronounced night vision difficulties compared to those with CSNB2, although these challenges are often not described as severe. While individuals with CSNB1 may struggle in very dark conditions, such as starlight, they regain functional vision quickly when light levels increase, as under full moonlight or artificial street lighting. However, patients with CSNB2 report minimal night vision issues, demonstrating relatively intact visual fields and better adaptation even under scotopic conditions.^[77]^ Cammack et al highlighted the psychological and social impact of night blindness by exploring the lived experiences of a patient with CSNB. The reluctance to disclose vision problems due to fear of social stigma often discourages participation in social activities and the adoption of coping strategies such as navigating using light reflections or point sources. These behaviors reflect the significant emotional and practical adaptations required to maintain independence and self-esteem.^[78]^ Furthermore, the World Health Organization confirms that night blindness limits essential daily activities, such as eating or finding objects after dusk, particularly in children and pregnant women, thereby exacerbating social and functional challenges.^[79]^ Together, these findings corroborate the profound yet variable impact of night blindness on QoL and call for tailored interventions to mitigate its effects.

The economic and social burdens of the aforementioned diseases are also considerable. Prem Senthil et al highlighted the financial strain linked to IRDs, noting that economic and social QoL domains were particularly impacted. Patients often face challenges related to employment, social interactions, and independence due to their vision loss. The study underscored the importance of addressing these broader QoL dimensions in clinical care and research.^[73]^

Furthermore, advances in gene therapy and other treatments for IRDs necessitate robust QoL assessment tools to evaluate patient-reported outcomes. McGuinness et al investigated the limitations of generic QoL instruments, such as the EQ-5D-5L, in capturing the full impact of vision impairment on patients' lives.^[76]^ Instead, vision-specific tools like the NEI VFQ-25 and disease-specific item banks, such as those developed by Prem Senthil et al, offer more precise insights into the QoL-related effects of IRDs.^[73]^

In conclusion, IRDs other than retinitis pigmentosa and Stargardt disease significantly reduce patients' QoL, with unique challenges posed by cone-rod dystrophies, LCA, Best disease, and choroideremia. These challenges span physical, psychological, social, and economic dimensions. Tailored interventions and the development of disease-specific QoL assessment tools are essential to address the multifaceted needs of patients experiencing these rare retinal conditions.

##  SUMMARY 

Posterior segment eye diseases significantly impact patients' QoL across physical, psychological, social, and economic dimensions. Conditions such as DR, AMD, optic neuropathy, and inherited retinal disorders cause significant visual impairment, which disrupts daily functioning, independence, and mental well-being. The economic burden of treatment and productivity loss will further exacerbate the challenges faced by patients. Effective management of these diseases requires a multidisciplinary approach integrating medical treatments, vision rehabilitation, and psychological support to address both clinical outcomes and the broader QoL impact. Emerging therapies, including gene and pharmacological treatments, hold promise for improved outcomes. Future research should focus on innovative interventions and patient-centered care strategies to enhance the overall well-being of individuals affected by posterior segment eye diseases.

##  AI Use Disclosure

Artificial intelligence applications were utilized for English language editing to improve clarity, grammar, and overall readability. However, the authors conducted all scientific content and interpretations without relying on AI tools.

##  Financial Support and Sponsorship

None.

##  Conflicts of Interest

None.
